# Prevalence and factors associated with diabetes-related distress in type 2 diabetes patients: a study in Hong Kong primary care setting

**DOI:** 10.1038/s41598-024-61538-w

**Published:** 2024-05-09

**Authors:** Man Ho Wong, Sin Man Kwan, Man Chi Dao, Sau Nga Fu, Wan Luk

**Affiliations:** Family Medicine and Primary Health Care Department, Kowloon West Cluster, Hospital Authority, Kowloon, Hong Kong

**Keywords:** Type 2 diabetes, Diabetes-related distress, Emotional burden, Chinese version of the diabetes distress scale (CDDS-15), Diabetes care, Psychology, Endocrine system and metabolic diseases

## Abstract

Diabetes-related distress (DRD) refers to the psychological distress specific to living with diabetes. DRD can lead to negative clinical consequences such as poor self-management. By knowing the local prevalence and severity of DRD, primary care teams can improve the DRD evaluation in our daily practice. This was a cross-sectional study conducted in 3 General Out-patient Clinics (GOPCs) from 1 December 2021 to 31 May 2022. A random sample of adult Chinese subjects with T2DM, who regularly followed up in the selected clinic in the past 12 months, were included. DRD was measured by the validated 15-item Chinese version of the Diabetes Distress Scale (CDDS-15). An overall mean score ≥ 2.0 was considered clinically significant. The association of DRD with selected clinical and personal factors was investigated. The study recruited 362 subjects (mean age 64.2 years old, S.D. 9.5) with a variable duration of living with T2DM (median duration 7.0 years, IQR 10.0). The response rate was 90.6%. The median HbA1c was 6.9% (IQR 0.9). More than half (59.4%) of the subjects reported a clinically significant DRD. Younger subjects were more likely to have DRD (odds ratio of 0.965, 95% CI 0.937–0.994, *p* = 0.017). Patients with T2DM in GOPCs commonly experience clinically significant DRD, particularly in the younger age group. The primary care clinicians could consider integrating the evaluation of DRD as a part of comprehensive diabetes care.

## Introduction

It is estimated that the prevalence of type 2 diabetes mellitus (T2DM) in adults in Hong Kong (HK) is approximately 10% of the population^1^. The Hospital Authority of Hong Kong provides public healthcare services to around 400,000 diabetic patients, with the General Out-patient Clinics (GOPCs) offering primary care to over 60% of these individuals^2^. People living with T2DM are affected by this chronic and progressive condition not only physically, but also emotionally. Diabetes-related distress (DRD) refers to the psychological distress specific to living with diabetes. It includes a wide range of emotions, such as feeling overwhelmed by the demands of self-management and restrictions. People with T2DM have to control diet, regularly do exercise and take medications^3^. Many of them may have fears of existing or future diabetes complications, concerns about hypoglycaemia and frustration with care providers^4^.

DRD involves emotional symptoms that may overlap with some psychological conditions, such as depression. However, a previous literature has demonstrated that DRD and depression are different constructs that need different assessment and management approaches^5^. Compared to depression, DRD is peculiar to the emotional distress caused by relentless self-management of diabetes and it does not imply underlying psychopathology. Also, DRD is more closely associated with diabetes-related behavioural and biomedical outcomes than depression. Particularly, it has been shown that DRD influences glycaemic control whereas the impact of depression appears to be equivocal^5–7^. Compared to depression, DRD is highly responsive to clinical intervention^4^. A systemic review has shown that interventions delivered by primary care clinicians, psychoeducation and motivational interviewing resulted in significant DRD reduction^8^.

DRD is prevalent among patients with T2DM, in which a meta-analysis demonstrated the overall prevalence of DRD was 36%^2^. Also, studies in China found that 42.5–77.2% of Chinese people with T2DM experienced DRD^9–12^. The occurrence of DRD may be influenced by age, gender, culture, type of diabetes, use of insulin, number of complications and duration of diabetes^13^. DRD can lead to negative clinical consequences as studies have shown that a high level of DRD was associated with poor self-management, suboptimal glycaemic control and poor quality of life^14–17^. The American Diabetes Association recommended that DRD should be routinely monitored, particularly when treatment targets are not met and/or at the onset of diabetes complications^18^. However, DRD is not assessed or recognized in most of the primary care practices in Hong Kong. Since the local prevalence and severity of DRD remain unknown, it is difficult to determine whether DRD assessment should be routinely included in local DM care.

The primary objective of this study was to study the proportion of clinically significant DRD among patients with T2DM in GOPCs in HK. The secondary objective was to identify the associated factors of DRD.

There are 2 hypotheses in this study. (1) The proportion of clinically significant DRD among patients with T2DM in GOPC in HK is common, which is at least 36%, according to existing literature. (2) There is a significant association of DRD with demographic and clinical parameters.

## Methodology

### Study design

This was a cross-sectional and prospective study conducted in three GOPCs in HK from 1 December 2021 to 31 May 2022. The three GOPCs include South Kwai Chung Jockey Club GOPC, Ha Kwai Chung GOPC and Cheung Sha Wan Jockey Club GOPC. The inclusion criteria were all adult Chinese patients, who had known diagnosis of T2DM and had at least two regular follow-ups for T2DM in the selected clinic in the past 12 months.

The exclusion criteria were patients diagnosed with type 1 diabetes, patients who had active follow-up of T2DM or were prescribed DM medications in Medicine Department specialist out-patient clinic, patients with known psychiatric illnesses who had active follow-up in either Psychiatry specialists or mental health services, patients who did not have diabetes related blood tests in the past 12 months from the study period, pregnant women, patients who did not understand written Chinese and mentally incapacitated persons.

A list of patients assigned with the International Classification of Primary Care (ICPC) code T90 (Diabetes; non-insulin-dependent) in the selected clinic was drawn from the Hospital Authority’s Clinical Data Analysis and Reporting System (CDARS) 2 weeks prior to the scheduled follow-up appointment with a corresponding appointment number. Up to 5 patients were selected from the list using random number table each day during the study period. A reminder was set in the computer system to identify those selected patients. The patients were invited and asked for consent to participate the study by the attending doctors. Information sheets about the study were given. Patients would complete the questionnaire individually and return it to the healthcare assistant in the clinic. Patients who refused to participate or give consent in this study were regarded as non-responders. Patients who had incomplete questionnaires or missing data were excluded from the statistical analysis. This study follows the principles of Declaration of Helsinki.

### Sample size

The sample size was calculated by using the sample size formula:

$${\text{n}} = \left( {{\text{Z}}^{{2}} \times {\text{ P }} \times \, \left( {{1} - {\text{P}}} \right)} \right)/{\text{e}}^{{2}} ,$$

where the desired precision was taken to be within 5% at 95% confidence interval.

- Z = value from standard normal distribution corresponding to desired confidence level (Z = 1.96 for 95% CI)
- P is expected true proportion
- e is desired precision (margin of error).

The expected proportion in the study population was set to be 36% based on the overall prevalence in the previous meta-analysis study^2^.

Therefore,

$${\text{n }} = \, \left( {{1}.{96}^{{2}} \times \, 0.{36 } \times \, 0.{64}} \right)/0.0{5}^{{2}} = { 355}$$

Assuming the response rate was 90%, the sample size was estimated to be 355/0.9 = 395 patients, which would round up to 400 patients. Thus, we would aim at recruiting at least 400 patients.

### Measurement

Diabetes Distress Scale (DDS) is one of the most commonly used and validated self-report measures to evaluate DRD internationally. The DDS is specific to patients with T2DM and provides a more comprehensive assessment to overcome the psychometric limitations of other measures such as Problems Areas in Diabetes (PAID) scale^2^. Another strength is that DDS also allows healthcare providers to identify the key sources of DRD^4^. The Chinese version of the Diabetes Distress Scale (CDDS-15) was validated in Hong Kong with consistent factor structure and good internal reliability (Cronbach’s alpha 0.902), which is specific for clinical use in Hong Kong Chinese type 2 diabetic patients^19^. There are 3 categories of CDDS-15, consisting of emotional burden (6 items), regimen- and social support- related distress (6 items), and physician-related distress (3 items)^19^. Each item was rated by patients using a 6-point Likert scale from 1 for “not a problem” to 6 for “a very serious problem.” The total mean item score was determined by adding the responses for all items and dividing by 15. Each subscale mean score was calculated by summing item responses in that subscale and dividing by the corresponding number of items. As reported by the study “When is diabetes distress clinically meaningful?: establishing cut points for the Diabetes Distress Scale”, an overall mean score ≥ 2.0 is considered clinically significant^17^. DRD was regarded as a dichotomous variable in this study, with subjects considered to have clinically significant DRD if CDDS-15 mean score ≥ 2.0.

We collected the data by using a printout questionnaire, consisting of three components: (1) The score of the CDDS-15; (2) demographic characteristics such as age, gender, education level, employment status, need of financial assistance to support basic living with Comprehensive Social Security Assistance (CSSA), living arrangement, and smoking status; (3) clinical parameters were obtained by reviewing participants’ medical records, including duration of T2DM, number of oral hypoglycaemic agent, use of insulin, latest Haemoglobin A1c (HbA1c) level, body mass index (BMI), diabetes complications and frequency of hypoglycaemic episodes in the past month. (see Appendix).

### Outcomes

The primary outcome was the proportion of DRD among patients with T2DM in the selected study centres. The secondary outcome was the associated factors of DRD including demographic characteristics and clinical parameters as mentioned above.

### Statistical analysis

The collected data was analyzed using the IBM Statistical Product and Service Solutions (SPSS) version 25 software. Qualitative variables were presented as frequencies and percentages. Quantitative variables were described as mean and standard deviation (SD), or median and interquartile range (IQR), as appropriate.

Pearson’s Chi-squared test was performed to compare the qualitative variables between participants without clinically significant DRD (DDS < 2) and participants with clinically significant DRD (DDS ≥ 2). Student’s *t-*test and Mann–Whitney U test was applied for quantitative variables with normal and non-normal distribution, respectively. When variables showed a *p*-value < 0.2 in the univariate analysis, they would be incorporated into the multivariate analysis. It was done to assure that all potentially associated variables were studied. Logistic regression analysis was used to adjust the confounding effect between variables and to identify the associated factors of DRD in those participants. Findings were considered statistically significant when the *p*-value < 0.05.

### Ethics approval and consent to participate

Informed consent in written form was obtained from all patients. The study was approved by the Hospital Authority Kowloon West Cluster Research Ethics Committee (KWC REC Reference: KW/EX-21-121(162-06)). The CDDS-15 questionnaire was granted permission for use in this study by American Diabetes Association (Permission Request Number: KL072021-MHW). This study follows the principles of Declaration of Helsinki.

## Results

### Patients’ demographic and clinical characteristics

We distributed 408 questionnaires, thirty-eight patients refused to participate in the study and the response rate was 90.6%. Eight questionnaires were found to have incomplete data and were discarded. Therefore, the total number of questionnaires included in the statistical analysis was 362.

Among the 362 participants, the mean age was 64.2 years old (SD 9.5) and male to female ratio was approximately 1:1. Fewer than 8% of participants (n = 27) had attained tertiary education. Approximately 40% of the participants (n = 146) were retired. The median HbA1c was 6.9% (IQR 0.9). The median duration of living with T2DM since diagnosis was 7.0 years (IQR 10.0). The mean BMI was 26.0 (SD 3.9). For the regimen type, approximately 90% of the participants (n = 324) were taking oral hypoglycaemic agents with or without insulin. The participants’ demographic and clinical characteristics were presented in Table 1.

**Table 1:** Demographic and clinical characteristics of patients analyzed.

Variables	All subjects (n = 362)	Without DRD (DDS < 2) (n = 147)	With DRD (DDS ≥ 2) (n = 215)	*p*-value^1^
Mean ± SD or median (IQR) or n (%)				
**Age (years)**	64.2 ± 9.5	66.3 ± 9.4	62.8 ± 9.4	< 0.001^
< 65	176 (48.6%)	57 (38.8%)	119 (55.3%)	0.002
≥ 65	186 (51.4%)	90 (61.2%)	96 (44.7%)	
**Gender**				
Male	182 (50.3%)	67 (45.6%)	115 (53.5%)	0.139
Female	180 (49.7%)	80 (54.4%)	100 (46.5%)	
**Education level**				
Primary school or below	138 (38.1%)	57 (38.8%)	81 (37.7%)	0.726
Secondary school	197 (54.4%)	81 (55.1%)	116 (54.0%)	
Tertiary education or above	27 (7.5%)	9 (6.1%)	18 (8.4%)	
**Employment status**				
Unemployed	66 (18.2%)	20 (13.6%)	46 (21.4%)	0.015
Employed	150 (41.4%)	55 (37.4%)	95 (44.2%)	
Retired	146 (40.3%)	72 (49.0%)	74 (34.4%)	
**Living arrangement**				
Live alone	37 (10.2%)	14 (9.5%)	23 (10.7%)	0.717
Live with family	325 (89.8%)	133 (90.5%)	192 (89.3%)	
**Currently on financial assistance with CSSA**				
Yes	29 (8.0%)	10 (6.8%)	19 (8.8%)	0.484
No	333 (92.0%)	137 (93.2%)	196 (91.2%)	
** Current smoker**				
Yes	35 (9.7%)	12 (8.2%)	23 (10.7%)	0.423
No	327 (90.3%)	135 (91.8%)	192 (89.3%)	
**BMI** **(kg/m**^2^**)**	26.0 ± 3.9	25.7 ± 3.7	26.2 ± 4.0	0.219^
Normal (BMI 18.5–22.9)	76 (21.0%)	36 (24.5%)	40 (18.6%)	0.177
Overweight or obese (BMI ≥ 23.0)	286 (79.0%)	111 (75.5%)	175 (81.4%)	
**Duration of DM (years)**	7.0 (10.0)	7.0 (11.0)	7.0 (8.0)	0.526^#^
< 5	126 (34.8%)	50 (34.0%)	76 (35.3%)	0.592
5–10	121 (33.4%)	46 (31.3%)	75 (34.9%)	
> 10	115 (31.8%)	51 (34.7%)	64 (29.8%)	
** HbA1c (%)**	6.9 (0.9)	6.8 (0.9)	7.0 (0.9)	0.163^#^
< 7.0	190 (52.5%)	84 (57.1%)	106 (49.3%)	0.142
≥ 7.0	172 (47.5%)	63 (42.9%)	109 (50.7%)	
**Number of OHA**				
0	38 (10.5%)	20 (13.6%)	18 (8.4%)	0.269
1	146 (40.3%)	56 (38.1%)	90 (41.9%)	
≥ 2	178 (49.2%)	71 (48.3%)	107 (49.8%)	
**Use of insulin**				
Yes	27 (7.5%)	9 (6.1%)	18 (8.4%)	0.424
No	335 (92.5%)	138 (93.9%)	197 (91.6%)	
** Regimen type**				
Lifestyle only	38 (10.5%)	20 (13.6%)	18 (8.4%)	0.111
OHA with or without insulin	324 (89.5%)	127 (86.4%)	197 (91.6%)	
**Hypoglycaemic episode in past month**				
Present	35 (9.7%)	12 (8.2%)	23 (10.7%)	0.423
Absent	327 (90.3%)	135 (91.8%)	192 (89.3%)	
** DM complication**				
Present	109 (30.1%)	42 (28.6%)	67 (31.2%)	0.598
Absent	253 (69.9%)	105 (71.4%)	148 (68.8%)	

^1^Statistically significant *p* values are in bold (*p* < 0.05).

*P*-values by Student’s *t* test, Chi-squared test, or Mann–Whitney U test as applicable. ^: by Student’s *t* test; ^#^: by Mann–Whitney U test. Those without labelling are analyzed by Chi-squared test.

CSSA = Comprehensive Social Security Assistance; BMI = body mass index; DM = diabetes mellitus; OHA = oral hypoglycaemic agent.

### Proportion of DRD

**Figure 1:**
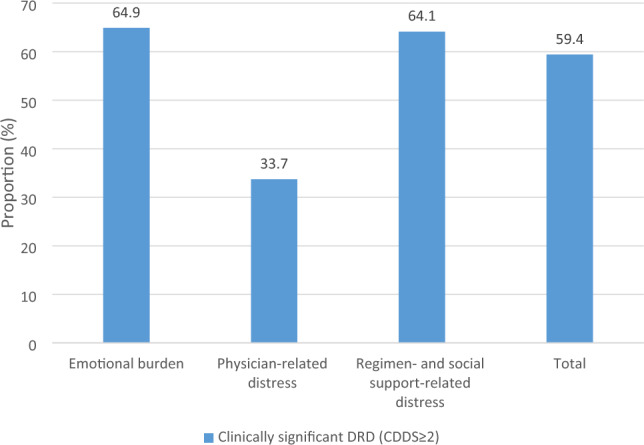
The proportion of clinically significant DRD among patients with T2DM in different subscales (n = 362).

A total of 59.4% of the study participants were found to have clinically significant DRD according to the total mean item score (DDS ≥ 2). Among the 3 subscales of DRD, emotional burden was observed in 64.9% of participants, followed by regimen- and social support-related distress (64.1%). Physician-related distress (33.7%) was relatively less affected. This is illustrated in Fig. 1.

### Factors associated with DRD

In the univariate analysis, age and employment status were found to be significantly associated with DRD (unadjusted *p* < 0.05). These factors, together with other variables with unadjusted *p* < 0.2 including BMI, HbA1c level and regimen type, were further analyzed in the multivariate logistic regression, as shown in Table 2. Only age was significantly associated with the occurrence of DRD among patients with T2DM, in which the adjusted odds ratio was 0.965 (95% CI 0.937–0.994, adjusted *p* = 0.017).

**Table 2:** Results of multivariate logistic regression on variables associated with clinically significant DRD.

Variables		Unadjusted			Adjusted		
		OR	95% CI	*p*-value	OR	95% CI	*p*-value
Age (years)	–	0.961	0.939–0.984	0.001	0.965	0.937–0.994	0.017
Gender	Male	referent			referent		
	Female	0.728	0.478–1.109	0.140	0.654	0.410–1.041	0.074
Employment status	Employed	referent			referent		
	Unemployed	1.332	0.715- 2.478	0.366	1.997	0.995–4.009	0.052
	Retired	0.595	0.374- 0.947	0.028	0.933	0.518–1.682	0.819
BMI (kg/m^2^)	Normal	referent			referent		
	Overweight or obese	1.419	0.853- 2.361	0.178	1.351	0.790–2.312	0.272
HbA1c (%)	< 7.0	referent			referent		
	≥ 7.0	1.371	0.899–2.091	0.143	1.314	0.844–2.045	0.227
Regimen type	Lifestyle only	referent			referent		
	OHA with or without insulin	1.724	0.878- 3.384	0.114	1.635	0.808–3.309	0.172

CI = confidence interval; OR = odds ratio.

## Discussion

In our study, 59.4% of patients with T2DM in the GOPC setting in HK suffered from clinically significant DRD. It is comparable to the studies in China with a reported prevalence 42.5–77.2%^9–12^. However, it is much higher than the overall prevalence 36% in the meta-analysis, in which the majority of the studies involved were from Western countries^2^. In Asia, the prevalence of DRD was reported to be 32%, 49%, and 53% in Singapore, Malaysia, and India, respectively^20–22^. The prevalence varies substantially across countries. This could be explained by the difference in the healthcare system, demographics, and cultural background.

Among the 3 subscales of DRD, the proportion of physician-related distress was the lowest in this study, which is similar to the findings in other studies^17,23^. Participants might not attribute their distress to physicians if they could obtain sufficient expertise and direction from physicians regarding their T2DM management. Nonetheless, healthcare professionals should pay more attention to the emotional side of diabetes care as more than 60% of subjects in this study had clinically significant emotional burden and regimen- and social support-related distress.

Our study showed that older age was associated with lower odds of DRD (OR 0.965). This is consistent with the results of other studies^24–26^. One study showed that the relation of DRD to psychological and behavioral outcomes is attenuated in older adults, regardless of the duration of T2DM^27^. One hypothesis is that older adults react less to stress because their previous experiences in coping with stress have led to better emotion regulation strategies^28^. On the other hand, younger patients usually have more responsibilities at work and family such as supporting their children and elderly family members^26^. These stressors can worsen the burden associated with the self-management of T2DM.

The HbA1c level was not significantly associated with DRD in our study. This is in line with the results of various international studies^2,16,23^. In contrast, a study conducted in a specialist clinic in HK using the CDDS-15 questionnaire showed that DRD had a positive relationship with HbA1c level^29^. The disparity may be explained by the difference in the healthcare setting and patients’ demographics. Also, only a minority of patients (7.5%) were prescribed insulin in the GOPC setting in our study, whereas 48% of the subjects were prescribed insulin in the specialist clinic in that study. In fact, there is mixed evidence in the literature regarding the relationship between glycaemic control and DRD^4^. Although DRD is modestly associated with poor glycaemic control, patients with good glycaemic control can also experience high DRD^4,16^. Achieving the HbA1c target may require intensive efforts that are potentially impacting other areas of their life such as social activities. This implies patients with T2DM may have an ongoing fear of disease complications or encounter challenges of self-management regardless of their latest glycaemic control.

The strengths of this study were that it was a multi-center study and there was a relatively high response rate. Measures such as invitations by healthcare providers could help reduce the number of non-responders. Moreover, it was one of the pioneer studies regarding DRD in the primary care setting in HK.

However, there are several limitations of this study. First, the use of a self-reported instrument in this study was influenced by social desirability bias. Physician-related distress might be underestimated in this study as patients might worry about negative effects on their treatment process if they declare a lack of confidence in the physician’s expertise in their diabetes management^30^. Second, the causality of the relationships could not be determined due to the study’s cross-sectional design. Further longitudinal studies are suggested to delineate causal relationships. Third, this study was conducted in three GOPCs only and there could be selection bias, therefore the study findings cannot be generalized to all patients with T2DM in HK. Fourth, it is important to acknowledge the restricted scope of this study on assessing other comorbidities such as hypertension and hyperlipidaemia. This study focused primarily on the clinical conditions directly associated with diabetes, including macrovascular and microvascular complications. Future studies could consider incorporating a boarder range of comorbidities to gain a more comprehensive understanding of the impact of diabetes-related distress. Lastly, as the study period coincided with the fifth wave of COVID-19 in HK, it could be a particularly stressful time for patients with T2DM to comply with their diet plan and exercise routine.

There are some clinical implications drawn from this study. Family physicians are on the frontlines responsible for the diagnosis and management of patients with T2DM and this study showed that a high proportion of patients with T2DM experience psychological distress. This finding alerts family physicians about the importance of a holistic approach in T2DM management. Regular evaluation of DRD by a self-reported instrument could be considered to incorporate with the annual assessment of T2DM in the GOPC setting. DRD does not typically disappear when left unaddressed, but DRD interventions do not require the expertise of a mental health professional^4^. In most cases, interventions offered by family physicians including motivational interviewing can help relieve DRD and thus improve the self-management of T2DM^4,8^. A practical guide on addressing DRD in clinical care is also available^4^. Further research on monitoring and addressing DRD in primary care in HK is warranted.

## Conclusion

The psychological component of diabetes is not routinely assessed in most of the primary care practices in HK. This study demonstrated that a high proportion of patients with T2DM in GOPCs experience clinically significant DRD. Younger age was identified as an associated factor. Evaluation of DRD is suggested to integrate as a part of comprehensive diabetes care in the primary care setting.

### Supplementary Information

Supplementary Information.

## Data Availability

The datasets used and/or analysed during the current study are available from the corresponding author on reasonable request.
