# Supplementary material for: Prevalence and factors associated with diabetes-related distress in type 2 diabetes patients: a study in Hong Kong primary care setting
Source: Sci Rep. 2024 May 9;14:10688. doi: 10.1038/s41598-024-61538-w (PMC11082166; doi:10.1038/s41598-024-61538-w)
Supplement: Supplementary file 1 — Supplementary Information. [file 41598_2024_61538_MOESM1_ESM.pdf]

SUPPLEMENTARY DATA

The 15-item version of the English and Chinese Diabetes Distress Scale (CDDS-15)

Directions: Living with diabetes can sometimes be tough. There may be many problems and hassles concerning diabetes and they can vary greatly in severity. Problems may range from minor hassles to major life difficulties. Listed below are 15 potential problems that people with diabetes may experience. Consider the degree to which each of the items may have distressed or bothered you DURING THE PAST MONTH and circle the appropriate number.

Please note that we are asking you to indicate the degree to which each item may be bothering you in your life, NOT whether the item is merely true for you. If you feel a particular item is not a bother or a problem for you, you would circle "1". If it is very bothersome to you, you might circle "6".

**Problems**	**Not a problem**		**Moderate problem**		**Serious problem**	
1. Feeling that diabetes is taking up too much of my mental and physical energy	1	2	3	4	5	6
2. Feeling that my doctor doesn't know enough about diabetes and diabetes care	1	2	3	4	5	6
3. Feeling angry, scared and /or depressed when I think about living with diabetes	1	2	3	4	5	6
4. Feeling that my doctor doesn't give me clear enough directions on how to manage my diabetes	1	2	3	4	5	6
5. Feeling that I am not testing my blood sugars frequently enough	1	2	3	4	5	6
6. Feeling that I am often failing with my diabetes regimen	1	2	3	4	5	6
7. Feeling that friends or family are not supportive enough of my self-care efforts (eg planning activities that conflict with my schedule, encouraging me to eat the "wrong foods").	1	2	3	4	5	6
8. Feeling that diabetes controls my life	1	2	3	4	5	6
9. Feeling that my doctors doesn't take my concerns seriously enough	1	2	3	4	5	6
10. Not feeling confident in my day-to-day ability to manage diabetes	1	2	3	4	5	6
11. Feeling that I will end up with serious long-term complications, no matter what I do	1	2	3	4	5	6
12. Feeling that friends or family doesn't appreciate how difficult living with diabetes can be	1	2	3	4	5	6

©2011 American Diabetes Association. Published online at http://care.diabetesjournals.org/lookup/suppl/doi:10.2337/dc10-1612/-/DC1

SUPPLEMENTARY DATA

Problems	Not a problem		Moderate problem		Serious problem	
13. Feeling overwhelmed by the demands of living with diabetes	1	2	3	4	5	6
14. Not feeling motivated to keep up my diabetes self-management	1	2	3	4	5	6
15. Feeling that friends or family don't give me the emotional support that I would like	1	2	3	4	5	6

Demographic information: Please circle the appropriate option.

1. Education level: Primary school or below / Secondary school / Tertiary education or above
2. Employment status: Unemployed / Employed / Retired
3. Living arrangement: Live alone / Live with family
4. Currently on Comprehensive Social Security Assistance: No / Yes
5. Hypoglycaemia episode in last 1 month: No / 1-2 times / 3 times or above

Thank you for completing the questionnaire!

Reprinted with permission from Rose Z.W. Ting, MRCP, Hairong Nan, PHD, Mandy W.M. Yu, MPH, Alice P.S. Kong, FRCP, Ronald C.W. Ma, FRCP, Rebecca Y.M. Wong, MSC, Kitman Loo, BSC, Wing-Yee So, MD , Chun-Chung Chow, FRCP, Gary T.C. Ko, MD , Yun-Kwok Wing, FRCPSYCH and Juliana C.N. Chan, MD : Diabetes-Related Distress and Physical and Psychological Health in Chinese Type 2 Diabetic Patients. https://doi.org/10.2337/dc10-1612 Copyright 2011 by the American Diabetes Association

** Items 12 and 15 in DDS-17 were removed after exploratory factor analysis to give rise to a new 15-item CDDS (CDDS-15).*

SUPPLEMENTARY DATA

The following is filled by attending doctor.

Patient's clinical information:

1. Gender: M / F
2. Age: _______
3. Smoking status: Non-smoker / Smoker / Ex-smoker
4. Duration of DM (year): ___________
5. Number of oral hypoglycaemic agent: ___________
6. Use of insulin: Yes / No
7. Latest HbA1c level (%): __________
8. BMI (kg/m^2): _________
9. Total DM complications: Please tick ✓

**Macrovascular**	**Microvascular**
Coronary heart disease __	Diabetic kidney disease (persistent microalbuminuria and/ or persistent decreased eGFR <60) __
Stroke __	Retinopathy (R1 or above) __
Peripheral arterial disease __	Neuropathy (VPT 25 or above) __

CDDS-15 Score and Subcategory

Total CDDS-15 Score:	a. Sum of 15 item scores. ______________<br>b. Divide by: _____15_______<br>c. Mean item score: ______________<br>Moderate distress or greater? (mean item score > 2) yes__ no__
A. Emotional Burden:	a. Sum of 6 items (1, 3, 8, 10, 11, 13) ______________<br>b. Divide by: _______6______<br>c. Mean item score: ______________<br>Moderate distress or greater? (mean item score > 2) yes__ no__
B. Physician-related Distress:	a. Sum of 3 items (2, 4, 9) ______________<br>b. Divide by: _______3______<br>c. Mean item score: ______________<br>Moderate distress or greater? (mean item score > 2) yes__ no__
C. Regimen- and Social Support-related Distress:	a. Sum of 6 items (5, 6, 7, 12, 14, 15) ______________<br>b. Divide by: _______6______<br>c. Mean item score: ______________<br>Moderate distress or greater? (mean item score > 2) yes__ no__

©2011 American Diabetes Association. Published online at http://care.diabetesjournals.org/lookup/suppl/doi:10.2337/dc10-1612/-/DC1
